# Association of Fine Particulate Matter (PM2.5) and Wet-Bulb Globe Temperature (WBGT) with Osteoporosis Incidence: A Nationwide Prospective Cohort Study

**DOI:** 10.7150/ijms.115460

**Published:** 2025-09-03

**Authors:** En-Che Chang, Fu-Wen Liang, Jiun-Chi Huang, Hao-Han Chang, Shu-Pin Huang, Szu-Chia Chen, Chih-Da Wu, Ya-Chin Huang, Jiun-Hung Geng

**Affiliations:** 1 Department of Urology, Kaohsiung Medical University Hospital, Kaohsiung Medical University, Kaohsiung, Taiwan.; 2 Department of Public Health, College of Health Sciences, Kaohsiung Medical University, Taiwan.; 3 Department of Medical Research, Kaohsiung Medical University Hospital, Kaohsiung Medical University, Taiwan.; 4 Center for Big Data Research, Kaohsiung Medical University, Taiwan.; 5 Department of Internal Medicine, Kaohsiung Municipal Siaogang Hospital, Kaohsiung Medical University, Kaohsiung, Taiwan.; 6 Division of Nephrology, Department of Internal Medicine, Kaohsiung Medical University Hospital, Kaohsiung Medical University, Kaohsiung, Taiwan.; 7 Department of Urology, School of Medicine, College of Medicine, Kaohsiung Medical University, Kaohsiung, Taiwan.; 8 Graduate Institute of Clinical Medicine, College of Medicine, Kaohsiung Medical University, Kaohsiung, Taiwan.; 9 Research Center for Environmental Medicine, Kaohsiung Medical University, Kaohsiung, Taiwan.; 10 Ph.D. Program in Environmental and Occupational Medicine, College of Medicine, Kaohsiung Medical University, Kaohsiung, Taiwan.; 11 Institute of Medical Science and Technology, College of Medicine, National Sun Yat-Sen University, Kaohsiung, Taiwan.; 12 Faculty of Medicine, College of Medicine, Kaohsiung Medical University, Kaohsiung, Taiwan.; 13 Department of Geomatics, College of Engineering, National Cheng Kung University, Tainan, Taiwan.; 14 National Institute of Environmental Health Sciences, National Health Research Institutes, Miaoli, Taiwan.; 15 Innovation and Development Center of Sustainable Agriculture, National Chung Hsing University, Taichung City, Taiwan.; 16 Research Center for Precision Environmental Medicine, Kaohsiung Medical University, Kaohsiung, Taiwan.; 17 Department of Preventive Medicine, Kaohsiung Medical University Gangshan Hospital, Kaohsiung Medical University, Kaohsiung, Taiwan.; 18 Department of Preventive Medicine, Kaohsiung Medical University Hospital, Kaohsiung Medical University, Kaohsiung, Taiwan.; 19 Department of Occupational & Environmental Medicine, Kaohsiung Medical University Hospital, Kaohsiung Medical University, Taiwan.; 20 Department of Urology, Kaohsiung Municipal Siaogang Hospital, Kaohsiung, Taiwan.

**Keywords:** Osteoporosis, Fine Particulate Matter, PM2.5, Wet-Bulb Globe Temperature, Ambient Temperature, Humidity

## Abstract

**Background:** Fine particulate matter (PM2.5) negatively impacts human health, contributing to cardiovascular, kidney, and lung diseases, as well as cancer. Emerging evidence suggests that PM2.5 exposure may also impair bone density, increasing osteoporosis risk. Ambient temperature and humidity interact with PM2.5, potentially influencing disease onset. However, most studies focus on Western populations or low-pollution environments and lack long-term follow-up data. This study investigated the association between PM2.5 exposure, wet-bulb globe temperature (WBGT), and osteoporosis in a large Taiwanese cohort.

**Methods:** Data from 19,981 participants in the Taiwan Biobank (TWB) were analyzed. PM2.5 exposure and WBGT were estimated using a Geo-AI-based ensemble mixed spatial model. Bone density was assessed using quantitative ultrasound, with osteoporosis defined as a T-score ≤ -2.5. Cox proportional hazards models assessed associations between PM2.5 exposure, WBGT, and osteoporosis risk.

**Results:** Among participants (65% women, mean age 51 years, BMI 24 kg/m²), 1,303 (6.5%) developed osteoporosis during a 43-month follow-up. Incidence rates by PM2.5 quartiles were: 8.4% (1st), 5.7% (2nd), 5.3% (3rd), and 6.6% (4th). High PM2.5 quartile exposure was associated with a 1.66-fold increased osteoporosis risk (HR: 1.66, 95% CI: 1.43-1.92, p < 0.001). Log-transformed PM2.5 exposure also showed significant risk (HR: 1.73, 95% CI: 1.02-2.95, p = 0.043). Higher WBGT (26-27°C) independently increased osteoporosis risk (HR: 1.49, 95% CI: 1.33-1.66, p < 0.001). WBGT further amplified risk among individuals with lower PM2.5 exposure.

**Conclusions:** PM2.5 exposure and elevated WBGT independently and interactively increased osteoporosis risk. Findings underscore the need for preventive strategies addressing environmental factors.

## Introduction

Osteoporosis is a systemic skeletal disorder marked by diminished bone mineral density (BMD), increased bone fragility, and deterioration of bone tissue microarchitecture. This imbalance in bone metabolism, where bone resorption surpasses bone formation, increases susceptibility to fractures. Osteoporosis is increasingly recognized as a critical public health concern, primarily due to its association with aging populations worldwide; the World Health Organization (WHO) projects that the global elderly population will reach 1.2 billion by 2025. The prevalence of osteoporosis varies by region, with the highest rate of 24.3% in Asia, followed by 16.7% in Europe, and 11.5% in the USA^1^. Osteoporosis and related fractures, both directly and indirectly, place a significant financial burden on the global economy. It has been estimated that the direct cost of hip fractures will increase in Asia from 9.5 billion United States dollars (USD) in 2018 to 15 billion USD in 2050, a 1.59-fold increase^2^.

Air pollution poses a significant global health threat, particularly in developing countries. Fine particulate matter (PM2.5) penetrates the respiratory system and enters the bloodstream, initiating tissue responses and systemic effects throughout the body^3^. PM2.5 appears to be linked to osteoporosis-related outcomes, however the evidence remains limited and inconsistent, requiring cautious interpretation due to study heterogeneity and risk of bias^4^, ^5^. PM2.5 exposure has been linked to a range of systemic disorders, including respiratory diseases, cardiovascular conditions, and impacts on both the nervous and renal systems^6^. Several studies have reported a significant correlation between PM2.5 exposure and osteoporosis. Zhang et al. found that long-term exposure to PM2.5 was associated with lower BMD scores and a higher risk of osteoporosis in rural Chinese populations^7^. Another study using UK Biobank data found that air pollution exposure, both alone and in combination with genetic factors, increased the risk of developing osteoporosis and fractures^5^. However, some studies have not found a significant association between PM2.5 exposure with osteoporosis and osteoporotic fractures^8^. Therefore, while there is growing evidence of a link between PM2.5 exposure and osteoporosis, inconsistencies in the findings and limitations in study designs underscore the need for further research to clarify this association.

Traditionally, air pollution has been measured using specialized instruments located at fixed monitoring stations, which are typically sparsely distributed in urban areas. Fixed monitoring stations can measure air pollution with high precision but low spatial resolution. Spatial modeling is essential for estimating air pollution variations, as understanding PM2.5 distribution is crucial for environmental studies to assess its health impacts^9^. Geospatial artificial intelligence (Geo-AI) offers a more precise and effective solution^10^, ^11^. By utilizing geographic information system (GIS) technologies and data from satellite imagery, aerial photography, and unmanned aerial vehicles, Geo-AI can accurately simulate the distribution of pollutant concentrations over large areas. This advanced modeling, powered by machine learning and ensemble learning techniques, allows for a more accurate and detailed understanding of the complex relationships between air pollution and surrounding land use, effectively modeling the concentration gradients of air pollution over large regions with over 90% accuracy. This methodology can strengthen the results of studies exploring the association between exposure to PM2.5 and various health outcomes, including the risk of osteoporosis.

Wet-bulb globe temperature (WBGT)^12^, ^13^, a composite measure of ambient temperature and humidity, is an important environmental factor that has been shown to influence many diseases. Climate change is driving an increase in WBGT, placing additional stress on populations and healthcare systems^14^. A strong correlation between extreme WBGT and increased mortality has been reported in previous studies, particularly among elderly populations, with higher rates of cardiorespiratory-related deaths during extreme weather conditions^15^. In addition, combined exposure to extreme WBGT and high PM2.5 has been associated with a significant increase in all-cause mortality by up 21.0%. Moreover, for cardiovascular and respiratory mortality, a previous study reported increases on days of co-exposure by 29.9% and 38.0%, respectively, exceeding the risks posed by either heat, humidity, or PM2.5 alone^16^. While the individual health risks of high WBGT and PM2.5 are well established, their combined effects on conditions such as osteoporosis remain underexplored.

This study aimed to investigate the combined effects of PM2.5 and WBGT on the risk of osteoporosis. We utilized data from a large, community-based cohort derived from the Taiwan Biobank (TWB), employing advanced Geo-AI technology to enhance the precision of our longitudinal analysis. By integrating air pollution and climate data, we sought to better understand how environmental factors, particularly heat stress and air pollution, interact to influence bone health over time.

## Methods

### Study population and design

**Figure 1:**
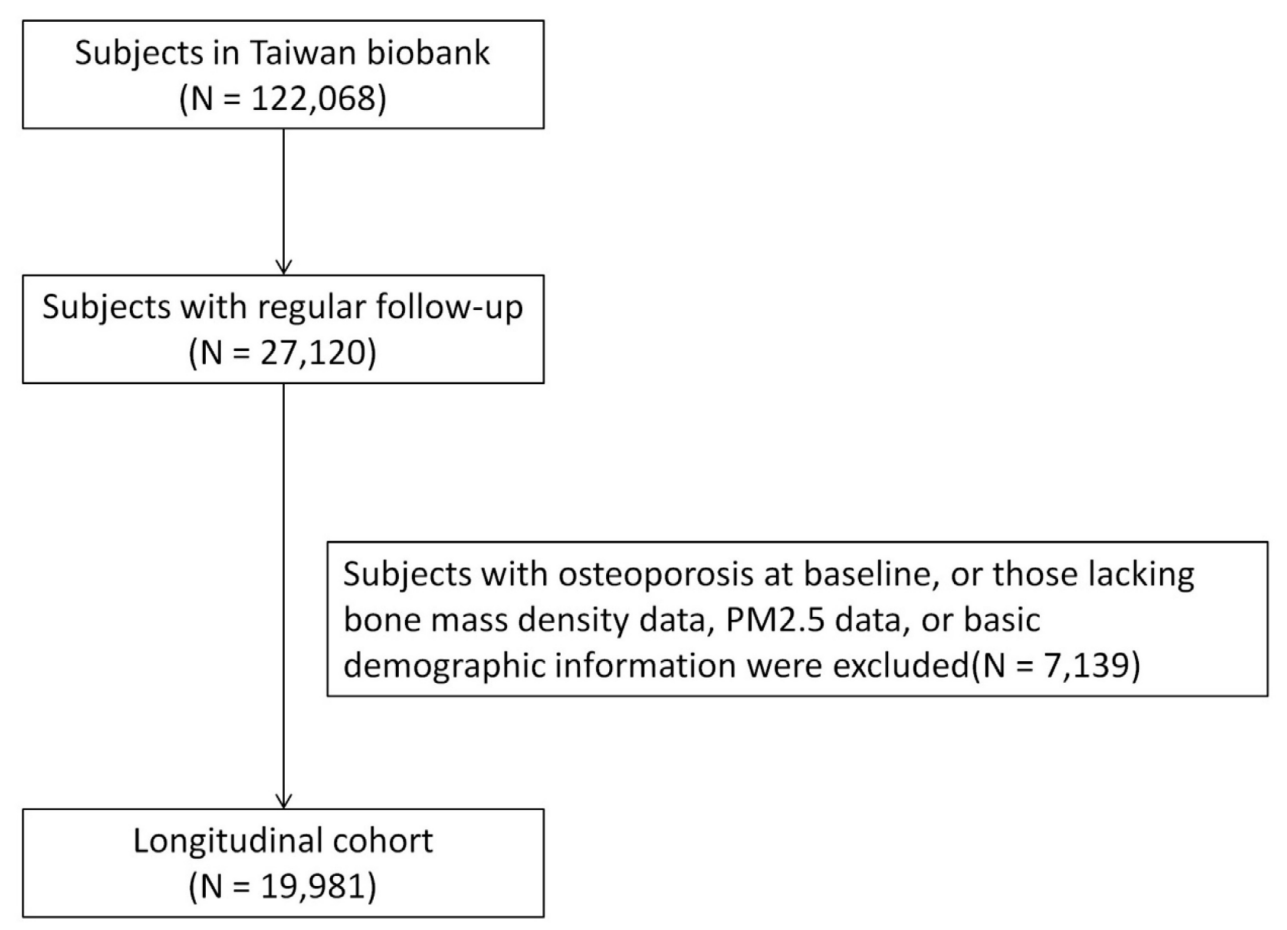
** Flowchart of study enrollment**.

Data were collected from the TWB between 2012 and 2018. Ethical approval was granted by the Ethics and Governance Council of the TWB and Institutional Review Board of Kaohsiung Medical University, under approval number KMUHIRB-E(I)-20210058, with the study period running from April 8, 2021 to March 31, 2026. Informed consent was obtained from all participants. The TWB database included 122,068 participants at the time of the study, of whom 27,120 had osteoporosis at baseline and regular follow-up data. After excluding participants with missing BMD, PM2.5 exposure, or basic demographic data, the remaining 19,981 participants were enrolled in the final analysis (**Figure 1**).

The participants provided detailed information on their residential address, age, sex, height, weight, smoking habits, alcohol consumption, educational status, and medical history, including hypertension, diabetes mellitus, dyslipidemia, and chronic kidney disease. They were followed for an average of 4 years to assess changes in their calcaneal T-scores. These data constituted a longitudinal cohort used for prospective analysis.

### PM2.5 and WBGT measurements using a Geo-AI system

**Figure 2:**
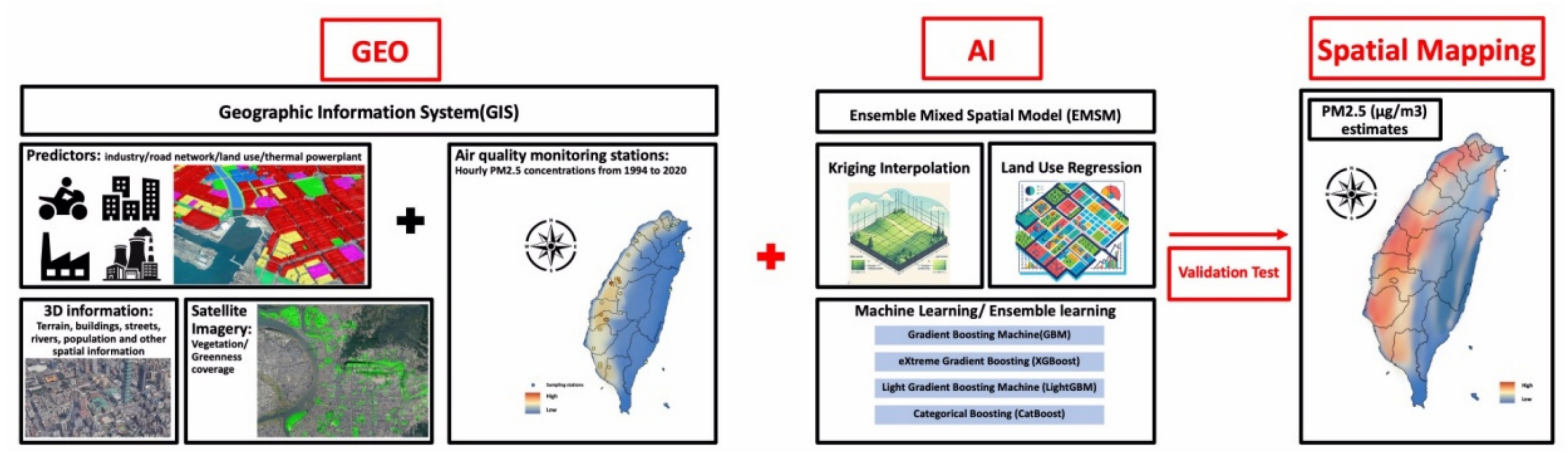
** The design flowchart of the novel Geo-AI system used in the present study.** We utilized a novel Geo-AI system, developed by Dr. Wu, to calculate PM2.5 and wet bulb globe temperature (WBGT). The system was designed by integrating Geographic Information System (GIS) data with artificial intelligence (AI) techniques and air pollutant levels, natural wet-bulb temperature, globe temperature and dry-bulb temperature from the Taiwan Environmental Protection Administration (EPA) to perform spatial mapping of long-term PM2.5 and WBGT variations and trends across Taiwan.

PM2.5 concentrations were measured using a novel Geo-AI system (**Figure 2**) designed by Dr. Wu, which integrates GIS data with AI techniques to enhance spatial analysis^9^. PM2.5 data were collected from 74 air quality monitoring stations across Taiwan maintained by the Taiwan Environmental Protection Administration (EPA) from 1994 to 2020. The Met-One BAM-1020 instrument (Met One Instruments, Inc., Grants Pass, Oregon, USA) was utilized for these measurements, detecting PM2.5 via beta-ray attenuation, with a detection range of 0 to 10 mg/m³ and a capture efficiency of >99.999% for particles with a 0.3 μm aerosol diameter. Diurnal variations in PM2.5 were accounted for by aggregating hourly data into daytime (6 a.m. to 6 p.m.) and nighttime (7 p.m. to 5 a.m.) concentrations. The Geo-AI system was used to model spatial and temporal patterns, incorporating satellite-derived variables, land-use data, and meteorological factors, allowing for a detailed estimation of long-term PM2.5 variations and trends across Taiwan^9^. Specifically, the ensemble mixed spatial model integrated land-use regression, satellite-based aerosol optical depth, and meteorological covariates with machine learning algorithms including random forest and gradient boosting. Model performance was evaluated using 10-fold cross-validation, demonstrating >90% predictive accuracy when compared with EPA monitoring station data^9^.

The Geo-AI system was also used to obtain WBGT data, integrating temperature and humidity using the formula: WBGT = 0.7*t_nw_* + 0.2*t_g_* + 0.1*t_a_*, where *t_nw_* is the natural wet-bulb temperature,* t_g_* is the globe temperature, and* t*_a_ is the dry-bulb temperature^11^, ^17^, ^18^. Hourly temperature data were obtained from 453 weather stations in Taiwan managed by the Central Weather Bureau from 2000 to 2020, including records from 427 automatic and 26 manual stations. Hourly WBGT values served as the dependent variable in a Land Use-Based Spatial Machine Learning (LBSM) model, aimed at predicting high spatial and temporal variations in WBGT across the main island of Taiwan at a high-resolution grid of 50 m × 50 m^11^.

To estimate outdoor PM2.5 exposure and WBGT data for each participant, we linked their residential addresses to the exposure data obtained through the Geo-AI system. We used 1 year of average exposure data from the index date to represent each participant's exposure level.

### BMD assessments and definition of osteoporosis

BMD was assessed using calcaneal quantitative ultrasound (Achilles InSight, GE, Madison Heights, USA). Quality assurance included cross-clinic calibration with phantoms and a review of a random sample of scans. T-scores (g/cm²) at the ankle were measured for each participant, calculated as the BMD minus the mean BMD of young adults, divided by the standard deviation (SD) of a normal young adult population. Osteoporosis was diagnosed when the T-score was ≤ -2.5 SD below the young adult reference value^19^.

### Statistical analysis

Participants were categorized into four groups based on PM2.5 exposure quartiles. Clinical characteristics were summarized as mean (± SD) for continuous variables such as age and body mass index (BMI), and as number (%) for categorical variables such as hypertension and diabetes mellitus. Differences between PM2.5 quartile groups were compared using ANOVA for continuous variables and chi-square tests for categorical variables. Multivariate Cox proportional hazards regression models were used to assess associations between PM2.5 exposure and the risk of developing osteoporosis. Hazard ratios (HRs) with 95% confidence intervals (CIs) were calculated to quantify the risks associated with PM2.5 exposure across quartiles, including log-transformed PM2.5 values, adjusting for potential confounders as specified in the results section. In addition, linear regression analysis was conducted to investigate associations between PM2.5 quartiles and decline in BMD during the follow-up period. Given prior evidence^12^, ^13^ suggesting that temperature and humidity may modify the effects of PM2.5 on health outcomes, the interaction between PM2.5 exposure and WBGT was explored through stratified analyses, with statistical significance set at a p-value < 0.05. All data analyses were performed using SPSS version 19 for Windows (IBM, Armonk, NY, USA).

## Results

### Baseline characteristics

The baseline characteristics of the study population were categorized into quartiles based on PM2.5 exposure levels (Q1: 11 μg/m^3^ to 26 μg/m^3^; Q2: 27 μg/m^3^ to 33 μg/m^3^; Q3: 34 μg/m^3^ to 38 μg/m^3^; Q4: 39 μg/m^3^ to 45 μg/m^3^) (**Table 1**). The data included age, BMI, sex distribution, smoking and alcohol use, exercise habits, educational status, and the prevalence of hypertension, diabetes, dyslipidemia, and chronic kidney disease. Among the 19,981 participants, 13,037 (65%) were women, and 6,944 (35%) were men, with an average age of 51 years and an average BMI of 24 kg/m². Significant differences were observed across quartiles in age, BMI, sex distribution, smoking and alcohol use, exercise habits, educational status, and hypertension history. Notably, participants in the highest quartile (Q4) were older (mean age of 52.26 years) compared to those in the lower quartiles (Q2 and Q3). A significant difference in sex distribution was also observed, with females comprising a larger proportion (68.6%) in the highest exposure quartile (Q4).

### Incidence of osteoporosis in the PM2.5 exposure quartile groups during follow-up

During a mean follow-up period of 43 months, 1,303 participants (6.5%) developed osteoporosis. The incidence rates in the PM2.5 exposure quartiles were as follows: Q1: 431 (8.4%), Q2: 277 (5.7%), Q3: 269 (5.3%), and Q4: 326 (6.6%) (**Table 2**). The follow-up period differed across the PM2.5 quartiles, with the shortest in Q4 (39 months) and longest in Q1 (46 months).

### Association between PM2.5 and incident osteoporosis

After adjusting for confounding factors, defined as variables with differences between groups and a p-value < 0.05 in **Table 1**, individuals in the highest PM2.5 exposure quartile (Q4) had a 1.655 times higher risk of developing osteoporosis compared to those in the lowest quartile (Q1) (HR: 1.655, 95% CI: 1.429 to 1.916, p-value < 0.001), as shown in **Table 3**. The analysis of log-transformed PM2.5 exposure also revealed a significant association with an increased risk of osteoporosis, with a HR of 1.732 (95% CI: 1.019 to 2.945, p-value = 0.043).

**Figure 3:**
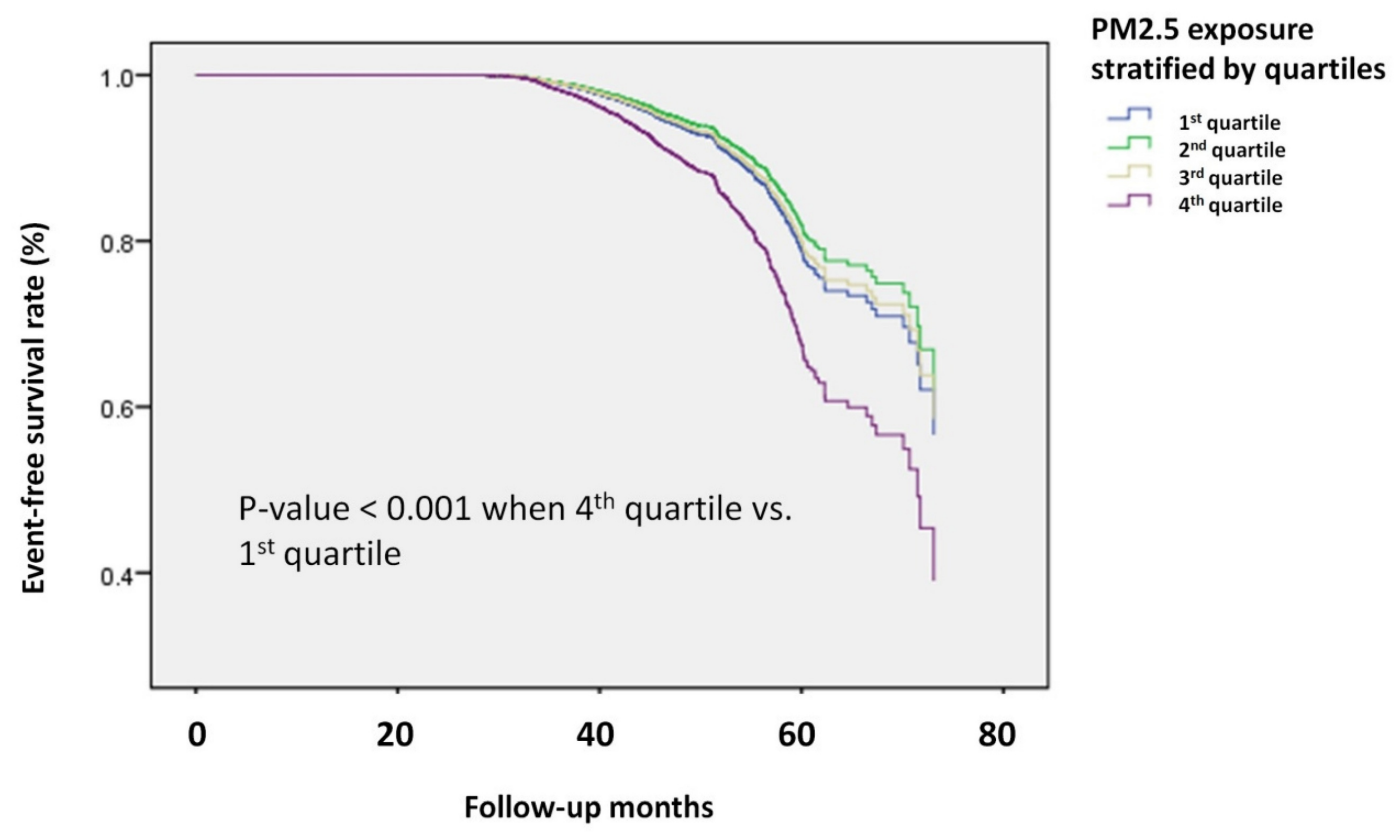
** Kaplan-Meier survival analysis of participants developing osteoporosis divided by PM2.5 quartiles.** The time to osteoporosis development was longer in the participants in the highest quartile of PM2.5 exposure compared to those in the lowest quartile. The Kaplan-Meier plot illustrates the incidence of osteoporosis development according to the quartiles of PM2.5 exposure among 19,981 participants with follow-up data.

In addition, Kaplan-Meier survival analysis (**Figure 3**) demonstrated a clear difference in the risk of osteoporosis between Q4 and Q1. These findings further strengthened the evidence linking higher PM2.5 exposure with an increased incidence of osteoporosis during follow-up.

Moreover, when we analyzed the decrease in BMD in each individual using linear regression analysis adjusted for confounding factors, the highest exposure group (Q4) had a greater decrease in T-scores over time compared to the lowest exposure group (Q1) (**Table 3**). Regarding the reduction in bone density, individuals in the highest quartile (Q4) had a beta value of -0.020 compared to those in Q1, indicating greater bone density loss.

### Association between WBGT and the risk of incident osteoporosis

We further examined the association between WBGT and the risk of developing osteoporosis in our cohort, categorized by median WBGT value (25°C). Participants exposed to high WBGT (26°C to 27°C) had a significantly increased risk of osteoporosis compared to those in the low WBGT group (16°C to 25°C), with an adjusted HR of 1.486 (95% CI: 1.331 to 1.659, p-value < 0.001). In addition, a one-unit increase in log-transformed WBGT was associated with a 29% higher risk of osteoporosis (HR: 1.291, 95% CI: 1.225 to 1.360, p-value < 0.001) (**Table 4**).

**Figure 4:**
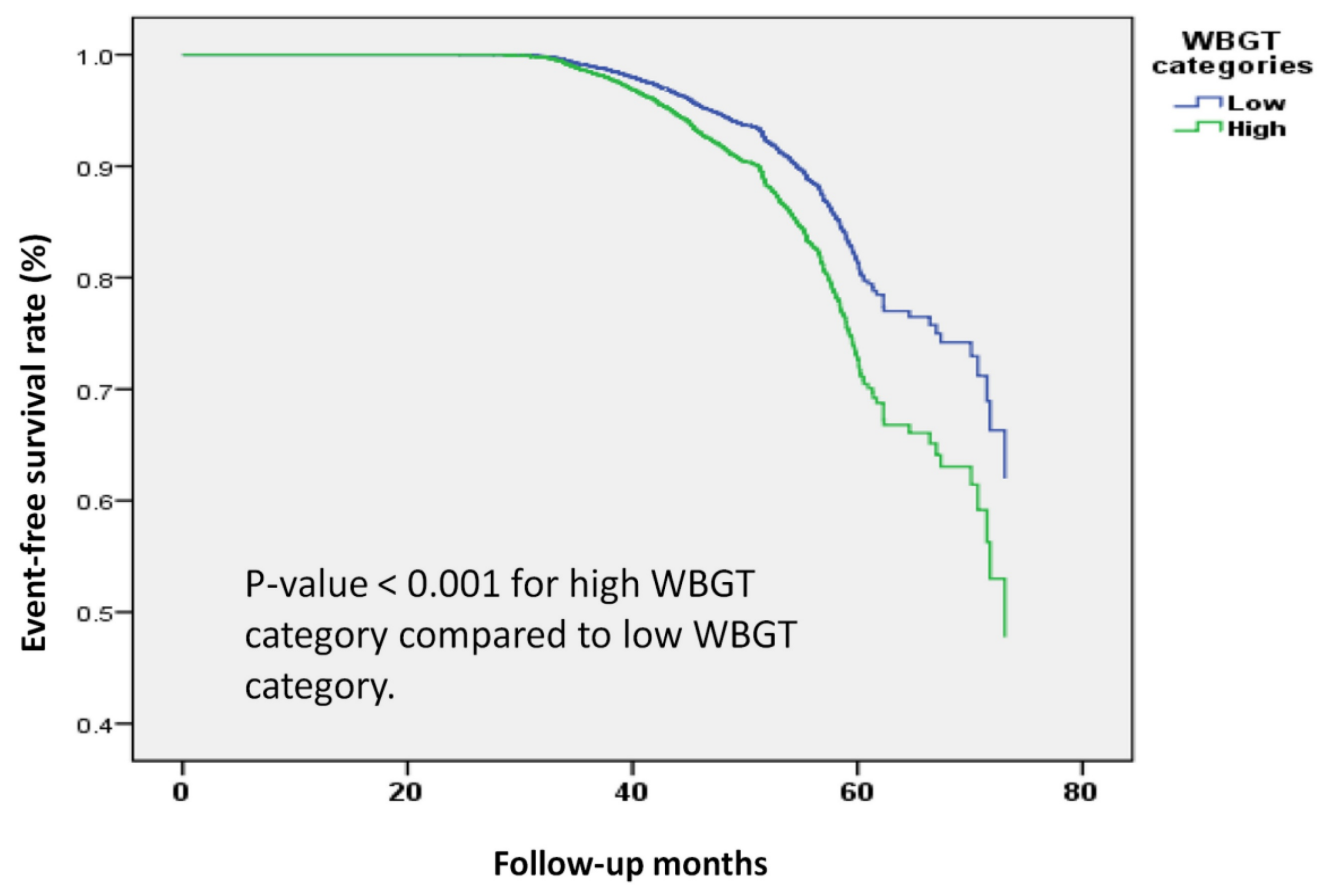
** Kaplan-Meier survival analysis of the participants developing osteoporosis by wet bulb globe temperature (WBGT) categories.** The time to osteoporosis development was longer in participants with high WBGT (26°C to 27°C) compared to those with low WBGT (16°C to 25°C). The Kaplan-Meier plot illustrates the incidence of osteoporosis according to WBGT categories among 19,981 participants with follow-up data.

In Kaplan-Meier survival analysis (**Figure 4**), a clear difference in the risk of osteoporosis was demonstrated between low and high WBGT groups. These findings further strengthened the evidence linking higher WBGT to an increased incidence of osteoporosis during follow-up.

### Effect of the interaction between PM2.5 exposure and WBGT on the risk of incident osteoporosis

Previous studies^12^, ^13^ have established that air pollutants are significantly influenced by temperature and humidity, leading to complex interactions in their health effects. To explore this further, we assessed the effect of the interaction between PM2.5 exposure and WBGT on the risk of developing osteoporosis, as presented in **Table 5**. WBGT was stratified into low (16°C to 25°C) and high (26°C to 27°C) categories based on the median value (25°C) specific to our cohort. Among participants in the lowest quartile of PM2.5 exposure (Q1), those exposed to high WBGT had a significantly greater risk of developing osteoporosis compared to those exposed to low WBGT (HR: 2.928, 95% CI: 2.211 to 3.878, p-value < 0.001). Similarly, participants in the third quartile of PM2.5 exposure (Q3) exposed to high WBGT also had an increased HR for incident osteoporosis (HR: 1.468, 95% CI: 1.131 to 1.905, p-value = 0.004). These results indicated that higher WBGT levels may amplify the effects of PM2.5 exposure on the risk of osteoporosis, underscoring the importance of considering environmental factors such as heat and humidity when evaluating bone health.

## Discussion

This study included approximately 20,000 participants with a 43-month follow-up period, and identified significant positive associations between PM2.5 exposure and WBGT with an increased risk of osteoporosis. Participants in the highest PM2.5 exposure quartile had a markedly higher HR of 1.655 compared to those in the lowest quartile. Similarly, participants in the high WBGT group had a higher HR of 1.486 compared to those in the low WBGT group. Notably, high WBGT further amplified the risk of osteoporosis, especially among the participants in the lowest PM2.5 quartile. To our knowledge, this is the first large-scale prospective study to demonstrate a significant link between air pollution and WBGT with incident osteoporosis, underscoring the importance of considering environmental factors when assessing bone health.

Our findings align with numerous studies that have identified a significant association between PM2.5 exposure and elevated osteoporosis risk. A study in Oslo^20^ reported an inverse relationship between air pollution and total body BMD, with a one SD increase in PM2.5 associated with a 33% higher odds (OR 1.33, 95% CI: 1.05-1.70) of low BMD. In Taiwan, long-term PM2.5 exposure has been linked to a higher risk of osteoporotic fractures in women, with an OR of 1.12 (95% CI: 1.03-1.22) per 10 μg/m³ increase in PM2.5^21^. Similarly, data from the UK Biobank^5^ demonstrated that air pollution, particularly when combined with genetic susceptibility, increased the risk of osteoporosis and fractures. In addition, Luo et al.^22^ found that chronic exposure to ambient air pollution, including PM2.5, was associated with notable bone mass loss among older adults.

Causal inference studies strengthen these epidemiological observations. Jiang et al. applied Mendelian randomization and reported a genetic instrument-based causal link between pollution and decreased bone health^23^. Similarly, Yu et al. identified genetic variants that modified the effect of pollution on osteoporosis risk, underscoring the interplay between environmental and genetic factors^5^. Collectively, these studies support the plausibility of our results and highlight the multifactorial nature of osteoporosis.

Moreover, landmark analyses from Prada et al. using the Women's Health Initiative cohort further established large-scale links between pollutants and osteoporosis. More recently, Prada et al.^24^ and Verbruggen et al.^25^ extended this work by identifying circulating metabolites and inflammatory mediators as potential intermediates, offering mechanistic explanations for how pollutants influence bone health. Building on this, Allen et al. synthesized recent findings in a comprehensive review, emphasizing both emerging consistency across studies and remaining gaps in understanding the pollution-osteoporosis relationship^26^. Our study extends this body of evidence by incorporating WBGT and applying a high-resolution Geo-AI exposure model, providing novel contributions beyond prior reviews.

However, not all studies have reported a clear association between PM2.5 exposure and osteoporosis risk. For example, Alvert^27^ found no increased risk of self-reported forearm fractures in a group exposed to PM2.5, suggesting that other factors may modulate this relationship. Similarly, Chiu et al.^21^ did not find a significant association between PM2.5 exposure (per 10-μg/m³ increase) and osteoporotic fractures in the overall population (adjusted OR: 1.02, 95% CI: 0.97 to 1.08) or among men (adjusted OR: 0.94, 95% CI: 0.86 to 1.02). These discrepancies may reflect differences in exposure duration, study design, and population characteristics. Variations in genetic background, environmental context, and lifestyle factors could also modulate susceptibility to PM2.5. Methodological issues, particularly the precision of exposure assessment and control for confounders, may further explain these inconsistencies. In our study, the use of a validated Geo-AI system allowed fine-scale exposure assessment, potentially offering more accurate estimates than studies relying on broader regional data.

Beyond epidemiology, experimental studies provide additional support. Kheirouri et al. observed mixed or even protective effects on bone mineral density in animals exposed to pollutants, highlighting variability in biological responses^28^. In contrast, Qinwen et al. demonstrated pollutant-induced bone loss in vivo and confirmed that inflammatory signaling directly altered osteoblast and osteoclast metabolism in vitro^29^. These findings underscore both the complexity and the biological plausibility of pollution-induced skeletal effects.

We also observed a novel interaction between WBGT and PM2.5, suggesting that these environmental factors may jointly compromise bone health. Elevated WBGT may increase metabolic demand, dehydration, and systemic stress responses, thereby amplifying the inflammatory and oxidative stress pathways triggered by PM2.5 exposure. Heat stress (as indexed by WBGT) can impair calcium metabolism, evident in reduced serum calcium and decreased intestinal absorption under sustained 35 °C exposure^30^, and promote osteoclast activation via inflammation, which can be mitigated by vitamin D supplementation^31^. Meanwhile, PM2.5 exposure has been linked to vitamin D deficiency and disrupted bone turnover, likely due to reduced UVB exposure in polluted areas^32^. Together, these stressors may synergistically amplify inflammatory and oxidative bone degradation pathways through calcium/vitamin D dysregulation, providing a plausible explanation for the observed synergy. Previous studies support temperature-related effects: a Norwegian cohort found that freezing temperatures increased fracture risk and mortality after hip fractures^33^, while a Chinese study reported higher osteoporotic fracture risk during colder periods, particularly among women^34^. In terms of co-exposure, a Californian study showed that extreme heat and PM2.5 synergistically increased all-cause, cardiovascular, and respiratory mortality^16^, whereas research in northern Thailand reported independent but not interactive effects^35^. Our findings thus provide new evidence that co-exposure to air pollution and temperature may jointly affect skeletal health, an issue of increasing importance in the context of climate change.

The adverse effects of PM2.5 on bone health are thought to be through several biological mechanisms. First, air pollutants trigger systemic inflammation by releasing proinflammatory cytokines. Liang et al. noted that air pollution exposure could activate inflammatory pathways, thereby disrupting the balance between osteoblasts and osteoclasts, and ultimately disturbing bone homeostasis^36^. Exposure to PM2.5 has been shown to activate immune cells, leading to the release of proinflammatory cytokines such as TNF-α and IL-6, both of which play a key role in bone remodeling and contribute to the development of osteoporosis^37^. Second, PM2.5 has been shown to induce oxidative stress by increasing reactive oxygen species​^38^, consequently disrupting the balance between bone-forming osteoblasts and bone-resorbing osteoclasts, and promoting bone resorption through mechanisms involving the nuclear factor erythroid-2-related factor 2 (Nrf2) pathway​^39^. Third, PM2.5 exposure has been reported to contribute to osteoporosis through endocrine disruption, particularly affecting estrogen, testosterone, and parathyroid hormone levels^40^. PM2.5 has also been shown to decrease estradiol, which is crucial for maintaining bone homeostasis, and estrogen deficiency has been shown to increase susceptibility to bone loss^41^. In men, PM2.5 lowers testosterone levels, leading to reduced bone density and strength^42^. In addition, PM2.5 has been shown to impact vitamin D and calcium metabolism, which disrupts the parathyroid hormone-regulated balance between bone resorption and formation, further accelerating osteoporosis^43^.

Beyond these well-established mechanisms, osteocytes, long overlooked compared to osteoblasts and osteoclasts, are now recognized as central regulators of skeletal remodeling. Acting as mechanosensors, osteocytes orchestrate bone turnover, and many therapeutic agents, including denosumab and sclerostin inhibitors, exert their effects through osteocyte-mediated pathways^44^. More recently, osteocytes have also been implicated in bone-cancer crosstalk and immune regulation^45^, ^46^, findings that align with the TNF-α-driven mechanisms discussed above.

This study has several limitations. First, osteoporosis was detected using quantitative ultrasound (QUS) instead of dual-energy X-ray absorptiometry (DXA), the gold standard for diagnosis. While DXA is accurate, it has limitations such as limited accessibility, radiation exposure, and high cost. In contrast, QUS is radiation-free, portable, and cost-effective. However, QUS has lower sensitivity (60-90%) and specificity (28-65%) compared to DXA^47^. Despite limited trials on QUS-diagnosed cases, previous studies have reported that a decrease in QUS measurements was correlated with a 1.5 to 2.5-fold increased risk of fractures, similar to DXA results^48^, ^49^. Second, we lacked data on medications or vitamin supplements that may have affected osteoporosis outcomes. Additionally, data on crucial factors such as indoor air pollution, time spent outside, parathyroid hormone levels, and vitamin D levels were also not included, which could have influenced the findings. Third, the focus on Taiwan, with its limited racial/ethnic diversity, may also limit the generalizability of the results. However, we adjusted for key confounders including age, BMI, smoking, alcohol use, physical activity, education, and chronic health conditions.

The strengths of this study are the large cohort size and comprehensive 4-year follow-up data, which increased the robustness of the results. In addition, we enhanced the methodology of air pollutant data collection by using a validated Geo-AI model to estimate population-level PM2.5 exposure, allowing for accurate determination of pollutant levels affecting diverse populations relative to monitoring stations. We also incorporated WBGT measurements into our exposure assessment, further improving the evaluation of environmental factors that contribute to bone health.

## Conclusion

This study is the first to utilize Geo-AI technology to investigate the effects of air pollution on osteoporosis in Taiwan. High PM2.5 exposure was identified as an independent risk factor for osteoporosis, with elevated WBGT levels further increasing the risk. These findings underscore the significant influence of fine particulate matter and environmental temperatures on bone health, highlighting the urgent need for targeted public health interventions.

## Figures and Tables

**Table 1:** Baseline characteristics of the study participants categorized by PM2.5 exposure quartiles (Q1 to Q4)

	All	PM2.5 Q1 (11 to 26)^#^	PM2.5 Q2 (27 to 33)^#^	PM2.5 Q3 (34 to 38)^#^	PM2.5 Q4 (39 to 45)^#^	
Characteristics	N = 19,981	N = 5,120	N = 4,862	N = 5,039	N = 4,960	*p-* value
Age (year)	51.20±10.36	51.76±10.20	50.49±10.69	50.28±10.16	52.26±10.28	<0.001
BMI (kg/m^2^)	24.15±3.60	24.21±3.57	24.20±3.63	24.17±3.58	24.01±3.60	0.019
Female, n(%)	13,037 (65.2)	3,310 (64.6)	3,115 (64.1)	3,207 (63.6)	3,405 (68.6)	<0.001
Ever smoking, n(%)	4,913 (24.6)	1,334 (26.1)	1,292 (26.6)	1,215 (24.1)	1,072 (21.6)	<0.001
Alcohol status, ever, n(%)	1,702 (8.5)	432 (8.4)	445 (9.2)	465 (9.2)	360 (7.3)	0.001
Exercise habits, yes, n(%)	9,329 (46.7)	2,450 (47.9)	2,110 (43.4)	2,268 (45.0)	2,501 (50.4)	<0.001
Educational status						<0.001
≤ Elementary	1,301 (6.5)	396 (7.7)	370 (7.6)	313 (6.2)	222 (4.5)	
Middle to high	8,800 (44.0)	2,412 (47.1)	2,004 (41.2)	2,178 (43.2)	2,206 (44.5)	
≥ College	9,880 (49.4)	2,312 (45.2)	2,488 (51.2)	2,548 (50.6)	2,532 (51.0)	
Hypertension, n(%)	2,716 (13.6)	742 (14.5)	603 (12.4)	682 (13.5)	689 (13.9)	0.020
Diabetes, n(%)	1,088 (5.4)	280 (5.5)	254 (5.2)	287 (5.7)	267 (5.4)	0.773
Dyslipidemia, n(%)	1,601 (8.0)	423 (8.3)	375 (7.7)	395 (7.8)	408 (8.2)	0.674
CKD	272 (1.4)	74 (1.4)	61 (1.3)	57 (1.1)	80 (1.6)	0.171

Abbreviations: BMI = Body Mass Index, CKD = Chronic Kidney Disease ^#^ PM2.5 in μg/m^3^

**Table 2:** Incidence of osteoporosis in the study participants during follow-up categorized by PM2.5 exposure quartiles (Q1 to Q4)

	All	PM2.5 Q1	PM2.5 Q2	PM2.5 Q3	PM2.5 Q4	
Characteristics	N = 19,981	N = 5,120	N = 4,862	N = 5,039	N = 4,960	*p-* value
Incident osteoporosis, n (%)	1,303 (6.5)	431 (8.4)	277 (5.7)	269 (5.3)	326 (6.6)	<0.001
Follow-up (months)	43±9	46±8	43±9	41±9	39±7	<0.001

**Table 3:** ** A.** Multivariate-adjusted hazard ratios for the development of osteoporosis; and **B.** Multivariate-adjusted hazard ratios for the decrease in bone mineral density (N = 19,981)

**A**		
Air pollutants	Adjusted hazard ratio (95% CI)	*p-*value
PM2.5 (4^th^ quartile vs 1^st^ quartile)	1.655 (1.429 to1.916)	<0.001
PM2.5 (3^rd^ quartile vs 1^st^ quartile)	0.940 (0.806 to 1.096)	0.429
PM2.5 (2^nd^ quartile vs 1^st^ quartile)	0.843 (0.724 to 0.981)	0.028
logPM2.5	1.732 (1.019 to 2.945)	0.043
		
**B**		
Air pollutants	Beta (95% CI)	*p-*value
PM2.5 (quartiles)	-0.020 (-0.029 to -0.005)	0.004

Covariates in the multivariable-adjusted model included age, sex, body mass index, smoking status, alcohol status, exercise habits, educational status and history of hypertension. Bold values denote statistical significance at the p-value < 0.05 level.

**Table 4:** Multivariate-adjusted hazard ratios for the development of osteoporosis by wet bulb globe temperature (WBGT) (N = 19,981)

WBGT categories	Incident osteoporosis cases / total subjects	Adjusted hazard ratio (95% CI)	*p-*value
Low (16°C to 25°C)	657 / 9,949 (6.6)	reference	-
High (26°C to 27°C)	646 / 10,032 (6.4)	**1.486 (1.331 to 1.659)**	<0.001
logWBGT	-	**1.291 (1.225 to 1.360)**	<0.001

Covariates in the multivariable-adjusted model included age, sex, body mass index, smoking status, alcohol status, exercise habits, educational status and history of hypertension. Bold values denote statistical significance at the p-value < 0.05 level.

**Table 5:** Multivariate-adjusted hazard ratios for the decrease of bone mineral density (N = 19,981)

Air pollutants	Adjusted hazard ratio (95% CI)	*p-*value
PM2.5 x WBGT		
Q1 x low	Reference	
Q1 x high	2.928 (2.211 to 3.878)	<0.001
Q2 x low	Reference	-
Q2 x high	1.166 (0.808 to 1.684)	0.412
Q3 x low	Reference	-
Q3 x high	1.468 (1.131 to 1.905)	0.004
Q4 x low	Reference	-
Q4 x high	0.892 (0.567 to 1.403)	0.622

Abbreviations: WBGT = Wet Bulb Globe Temperature. Covariates in the multivariable-adjusted model included age, sex, body mass index, smoking status, alcohol status, exercise habits, educational status and history of hypertension.
